# A Novel Mitochondrial-Related Nuclear Gene Signature Predicts Overall Survival of Lung Adenocarcinoma Patients

**DOI:** 10.3389/fcell.2021.740487

**Published:** 2021-10-25

**Authors:** Xiangwei Zhang, Wei Dong, Jishuai Zhang, Wenqiang Liu, Jingjing Yin, Duozhi Shi, Wei Ma

**Affiliations:** ^1^Department of General Thoracic, Shandong Provincial Hospital Affiliated to Shandong First Medical University, Jinan, China; ^2^Department of General Thoracic, Feicheng Hospital Affiliated to Shandong First Medical University, Feicheng, China; ^3^Department of General Thoracic, Shenxian County People’s Hospital of Shandong Provincial Group, Liaocheng, China; ^4^Lifehealthcare Clinical Laboratories, Hangzhou, China

**Keywords:** lung adenocarcinoma, nuclear mitochondrial genes, risk score, overall survival, signature

## Abstract

**Background:** Lung cancer is the leading cause of cancer-related death worldwide, of which lung adenocarcinoma (LUAD) is one of the main histological subtypes. Mitochondria are vital for maintaining the physiological function, and their dysfunction has been found to be correlated with tumorigenesis and disease progression. Although, some mitochondrial-related genes have been found to correlate with the clinical outcomes of multiple tumors solely. The integrated relationship between nuclear mitochondrial genes (NMGs) and the prognosis of LUAD remains unclear.

**Methods:** The list of NMGs, gene expression data, and related clinical information of LUAD were downloaded from public databases. Bioinformatics methods were used and obtained 18 prognostic related NMGs to construct a risk signature.

**Results:** There were 18 NMGs (*NDUFS2*, *ATP8A2*, *SCO1*, *COX14*, *COA6*, *RRM2B*, *TFAM*, *DARS2*, *GARS*, *YARS2*, *EFG1*, *GFM1*, *MRPL3*, *MRPL44*, *ISCU*, *CABC1*, *HSPD1*, and *ETHE1*) identified by LASSO regression analysis. The mRNA expression of these 18 genes was positively correlated with their relative linear copy number alteration (CNA). Meanwhile, the established risk signature could effectively distinguish high- and low-risk patients, and its predictive capacity was validated in three independent gene expression omnibus (GEO) cohorts. Notably, a significantly lower prevalence of actionable EGFR alterations was presented in patients with high-risk NMGs signature but accompanied with a more inflame immune tumor microenvironment. Additionally, multicomponent Cox regression analysis showed that the model was stable when risk score, tumor stage, and lymph node stage were considered, and the 1-, 3-, and 5-year AUC were 0.74, 0.75, and 0.70, respectively.

**Conclusion:** Together, this study established a signature based on NMGs that is a prognostic biomarker for LUAD patients and has the potential to be widely applied in future clinical settings.

## Introduction

Mitochondria are complex organelles of bioenergetic, biosynthetic, and signaling that are correlated to several diseases, including cardiovascular diseases, neurological disorders, and metabolism disorders (Vyas et al., 2016; Genovese et al., 2020). In addition, recently, mitochondria are demonstrated to participate in multiple aspects of tumor formation and progression (Hsu et al., 2016; Bonora et al., 2021). The mechanisms of mitochondria involved in tumorigenesis have been extensively studied (Nunes et al., 2015), and some specific nuclear mitochondrial genes (NMGs) were regarded as the potential targets for the development of the next generation of cancer therapeutics (Su et al., 2016; Zong et al., 2016).

mtDNA is the own genome of mammalian mitochondria, 16.5- kb double-stranded circular DNA, which encodes a total of 13 proteins that are all core components of oxidative phosphorylation (OXPHOS). However, all remaining mitochondrial functions proteins (∼1,300) are encoded in the nuclear DNA (nDNA). Following their translation, they are imported into the organelle through the import machinery instead (Anderson et al., 1981; DiMauro and Schon, 2003; Schon and Przedborski, 2011). Thus, the mitochondrial function requires cooperation between the nuclear and mitochondrial genomes. Mitochondrial genome instability and mitochondrial dysfunction are novel markers for cancer (Wang et al., 2015; Idaghdour and Hodgkinson, 2017). Mitochondrial dysfunction, caused by smoking or other factors, plays a vital role in the carcinogenesis of multiple cancers, especially lung cancer (Cloonan et al., 2020). The dysfunction of mitochondria may shift the energy production from mitochondria-mediated oxidative to glycolysis, which in turn increases the cell metabolism and activity (Chen et al., 2021). Meanwhile, mitochondrial dysfunction causes ROS outbreak, which mediates epithelial-mesenchymal transition and cell invasion in lung cancer via coordinate intracellular signal transduction (Nunes et al., 2015; He et al., 2016; Han et al., 2018). mtDNA variations have been frequently suggested to serve as initiators for a variety of tumors, such as prostate cancer (Hopkins et al., 2017), LUAD (Yuan et al., 2015), breast cancer (Weerts et al., 2018), pancreatic cancer (Lam et al., 2012), gynecological malignancies (Guerra et al., 2014), and acute myeloid leukemia (Reznik et al., 2016; Tyagi et al., 2018). Contrary to the increased study on the association of mtDNA alterations and cancer, fewer studies have shed light on the role of NMGs on cancer. The germline or somatic alteration in nuclear mitochondrial complex II genes encoding SDH subunits (*SDHB*, *C*, and *D*) were found associated with pheochromocytomas and paragangliomas (Gimm et al., 2000; van Nederveen et al., 2007; Bardella et al., 2011). Similarly, one study found that the NMGs *NDUFS1* and *NDUFS8* (encoding subunits of mitochondrial complex 1) had significant prognostic power in the patients with non-small cell lung cancer (NSCLC) by analyzed immunohistochemical staining and RNA expression data (Su et al., 2016). Alterations in the genes mentioned above have also been detected in patients with mitochondrial disease, such as mitochondrial complex I deficiency and mitochondrial respiratory chain deficiency (Bourgeron et al., 1995; Loeffen et al., 1998; Björkman et al., 2015). Therefore, we examined the hypothesis that the NMGs may have significant predictive value in the identification of high-risk cancer patients with poor overall clinical survival.

Lung cancer is the leading cause of cancer-related mortality worldwide causing over 1.7 million deaths annually (Bray et al., 2018), of which LUAD are the major subtypes. In the present study, we investigate the NMGs associated with LUAD survival by bioinformatics analysis, following univariate Cox regression analyses to develop an NMGs signature to provide new clues for improving the diagnosis and treatment of patients with LUAD.

## Materials and Methods

### Data Sources

The data of gene expression and corresponding clinical of LUAD were retrieved from cbioportal (TCGA, Firehose Legacy) and Gene Expression Omnibus (GEO) (GSE13213, GSE42127, and GSE72094) database. TCGA-LUAD dataset was chosen as the training dataset, and the other three datasets were selected as external validation. The last of NMGs was downloaded from MITOMAP (Last update: January 2018)^1^ and was detailed in Supplementary Table 1.

### Construction and Evaluation of a Survival-Related Nuclear Mitochondrial Genes Signature

First, univariate Cox regression analysis was used to identify prognosis-related NMGs (*P* < 0.05). Then, the least absolute shrinkage and selection operator (LASSO)-penalized Cox regression analysis was performed to build a prognostic model with the “glmnet” R package. The risk score calculation was based on the expression level of the normalized gene and regression coefficient of the corresponding gene, which was as follows: risk score = sum (expression level of each gene × coefficient of corresponding genes).

The patients in the training and validation cohorts were grouped into high- or low-risk groups based on the median risk score. Kaplan-Meier (KM) analysis was performed to compare the survival differences between the high- and low-risk groups using the R package. In addition, the area under the curve (AUC) was calculated to measure the prognostic capability of the NMGs signature, and the nomogram was drawn by the R package.

Multivariate Cox regression analyses were performed to determine the prognostic values for the signature and some clinical features. R package “rms” was used to draw nomograms and calibration curves.

### Gene Set Enrichment Analysis

To explore biological processes in the high-risk and low-risk groups, GSEA was performed by ClusterProfiler package in R studio. The HALLMARK gene sets and Kyoto Encyclopedia of Genes and Genomes (KEGG) gene sets from the Molecular Signatures Database (MSigDB)^2^ were used.

### Genomic Mutations and Tumor Microenvironment Analysis

To compare the differences in genomic mutations between high and low risk groups, mutation profiles were analyzed and visualized using the “maftools” R. Meanwhile, transcriptome profiles from the TCGA cohort were used to identify the immune cell fractions of 22 distinct leukocyte subsets by the CIBERSORT tool.

### Statistical Analyses

Fisher’s exact test was applied to compare the difference in proportions between the high- and low-risk groups. R software was used to perform statistical analysis and all statistical results with *p*-values < 0.05 were considered statistically significant.

## Results

### Baseline Characteristics of Lung Adenocarcinoma Patients Included in This Study

In this study, TCGA-LUAD, which included 502 samples, was applied as the discovery dataset. In addition, GSE13213, GSE42127, and GSE72094 databases, which contained 117, 133, and 420 LUAD patients, were used as the validation cohort. The details of baseline characteristics are shown in Table 1.

**TABLE 1:** Baseline characteristics of the patients in this study.

**Characteristics**	**TCGA-LUAD**	**GSE13213**	**GSE42127**	**GSE72094**
N	502	117	133	420
Age	65.32 ± 9.95	60.68 ± 10.17	65.76 ± 10.29	69.25 ± 9.3
**Gender**				
Male	231	60	68	188
Female	271	57	65	232
**Smoking**				
Yes	333	61	–	320
No	169	56	–	31
**Neoplasm disease stage**				
Stage I	269	79	89	–
Stage II	120	13	22	–
Stage III	80	25	20	–
Stage IV	25		1	–
**T stage**				
T1	168	54	–	–
T2	268	50	–	–
T3	45	8	–	–
T4	18	5	–	–
**N stage**				
N0	325	87	–	–
N1	95	8	–	–
N2	69	22	–	–
N3	2	0	–	–
**M stage**				
M0	334	117	–	–
M1	24	0	–	–
Fev1_fvc_ratio_prebroncholiator	79.72 ± 18.45	–	–	–
Fev1_fvc_ratio_postbroncholiator	78.26 ± 20.99	–	–	–

*T stage, tumor stage; N stage, lymph node stage; M stage, long-distant metastasis stage.*

### Construction of a Nuclear Mitochondrial Genes Signature

**FIGURE 1:**
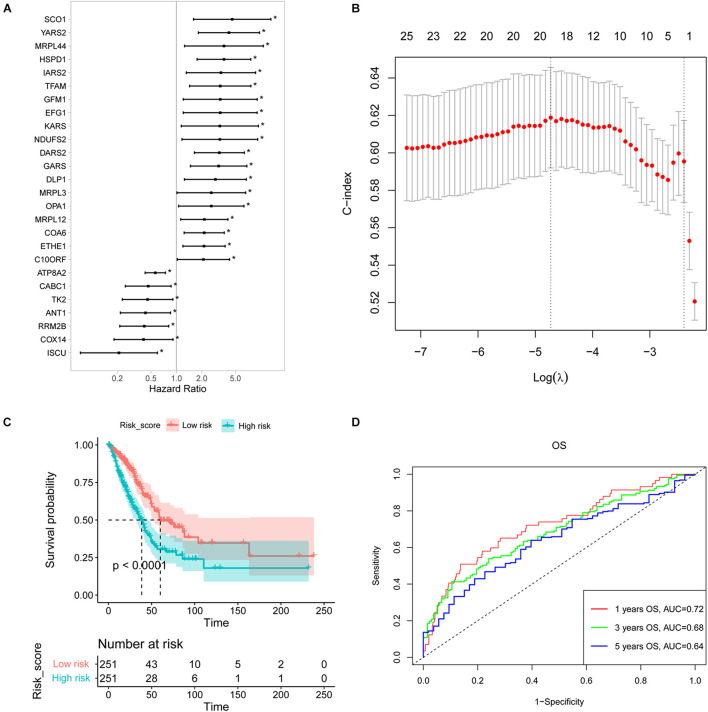
Construction of a survival-related signature based on nuclear mitochondrial genes (NMGs). **(A)** The forest plot of the associations between the expression levels of 26 genes and overall survival in LUAD patients. Hazard ratios (HR), *P*-value, and corresponding 95% confidence intervals were calculated by univariate Cox regression analysis. **(B)** The result of LASSO regression analysis. The vertical dotted lines were drawn at the optimal values by using the maximum and lambda.min criteria. **(C)** Kaplan-Meier OS curves for patients with high- and low-risk NMGs signature in the TCGA cohort. **(D)** The ROC curves for 1-, 3-, and 5-year survival of established NMGs signature. OS, overall survival; LASSO, the least absolute shrinkage and selection operator; ROC, receiver operating characteristic. *P < 0.05.

The patients from TCGA were used as a training cohort to identify an NMGs signature. A univariate Cox regression analysis was conducted, and 26 survival-related NMGs (Figure 1A) from a total of 146 NMGs were identified (*P* < 0.05) (Supplementary Table 1). Among the 26 genes, the hazard ratio (HR) of seven genes (*ATP8A2*, *CABC1*, *TK2*, *ANT1*, *RRM2B*, *COX14*, and *ISCU*) was less than 1, while the HR of the remaining 19 genes was more than 1, which indicated that the 7 genes were associated with a poor prognosis in LUAD patients.

Then we established a risk score model with these identified 26 survival-related NMGs using LASSO-based Cox regression analysis and 18 NMGs were selected (Figure 1B). The risk score was calculated using the following formula:


riskscore=(0.1738×EXP)NDUFS2+(-0.4258×EXP)ATP8⁢A2+(0.4404×EXP)SCO1+(-0.7904×EXP)COX14+(0.6436×EXP)COA6+(-0.0686×EXP)RRM2⁢B+(0.1818×EXP)TFAM+(0.2117×EXP)DARS2+(0.0779×EXP)GARS+(0.4276×EXP)YARS2+(0.3687×EXP)EFG1+(0.0001×EXP)GFM1+(-0.8567×EXP)MRPL3+(0.1498×EXP)MRPL44+(-0.8863×EXP)ISCU+(-0.4291×EXP)CABC1+(0.3229×EXP)HSPD1+(0.5369×EXP)ETHE1.

Using the median risk score, we classified the patients from the TCGA cohort into high-risk and low-risk groups. The Kaplan-Meier curve indicated that the patients in the low-risk group exhibited a longer survival time than those in the high-risk group [median overall survival (OS], 60.12 vs. 38.47 months, *P* < 0.0001, Figure 1C). Furthermore, the 1-, 3-, and 5-year OS predictions for LUAD patients and all the calibrated curves were well-fitted with AUCs of 0.72, 0.68, and 0.64, respectively (Figure 1D).

**FIGURE 2:**
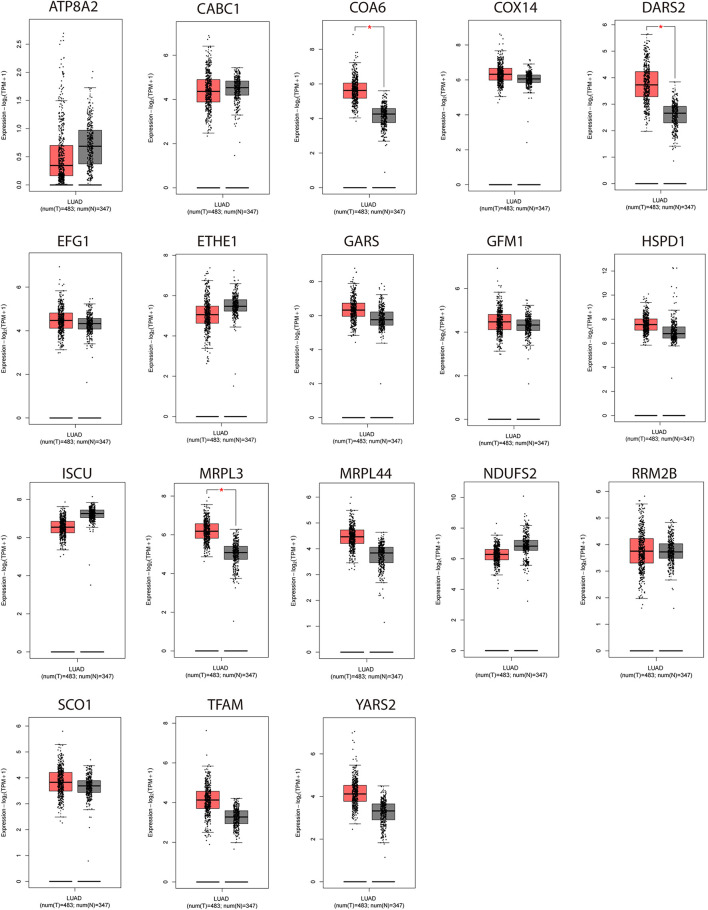
Expression analyses of the 18 genes (*NDUFS2, ATP8A2, SCO1, COX14, COA6, RRM2B, TFAM, DARS2, GARS, YARS2, EFG1, GFM1, MRPL3, MRPL44, ISCU, CABC1, HSPD1, ETHE1*) in LUAD samples and normal samples. *P < 0.05.

We analyzed the expression of the 18 NMGs in LUAD samples compared to normal samples using GEPIA to compile Genome Tissue Expression (GTEx) and TCGA datasets. The 18 genes were found to be expressed in both tumor and normal tissues, of which *COA6*, *DARS2*, and *MRPL3* were significantly more expressed in tumor tissues (*P* < 0.05, Figure 2).

### 18 Nuclear Mitochondrial Genes Signature Validation in Three Gene Expression Omnibus Cohorts

**FIGURE 3:**
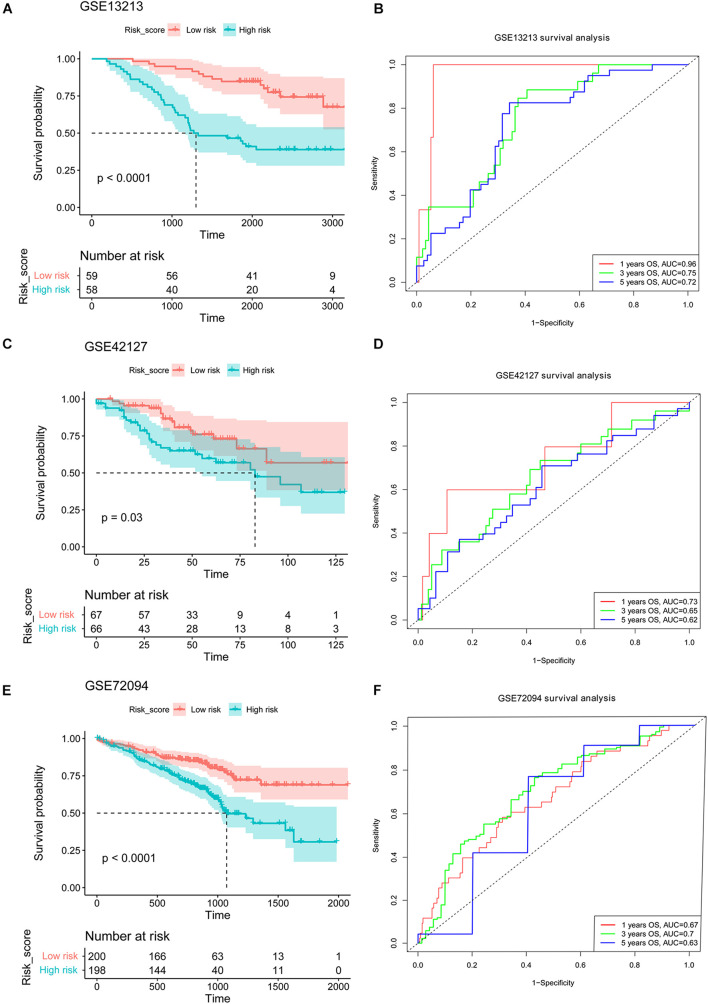
Validation of the established NMGs signature. Kaplan-Meier OS curves for patients assigned to high- and low-risk groups based on the risk score in the GSE13213 **(A)**, GSE42127 **(C)**, and GSE72094 **(E)** cohort. The ROC curves of 1-, 3-, and 5-year OS in the GSE13213 **(B)**, GSE42127 **(D)**, and GSE72094 **(F)** cohort. NMGs: nuclear mitochondrial genes; OS: overall survival; ROC: receiver operating characteristic.

We assessed the performance of established NMG’s signature using the three independent validation cohorts (GSE13213, GSE42127, and GSE72094) from the GEO. Based on the median value of risk score calculated as described above, the patients in the GSE13213, GSE42127, and GSE72094 were assigned to the high-risk and low-risk groups, respectively. The results were satisfactory that the patients in the low-risk group in all three validation cohorts had better survival than those in the high-risk group (all *P* < 0.05, Figures 3A,C,E). The receiver operating characteristic (ROC) curve for 1-, 3-, and 5-year OS predictions suggested that the model possessed predictive accuracy with AUCs above 0.600 (Figures 3B,D,F).

### Multivariate COX Regression Analyses Regarding Overall Survival in the TCGA-LUAD Cohort

**FIGURE 4:**
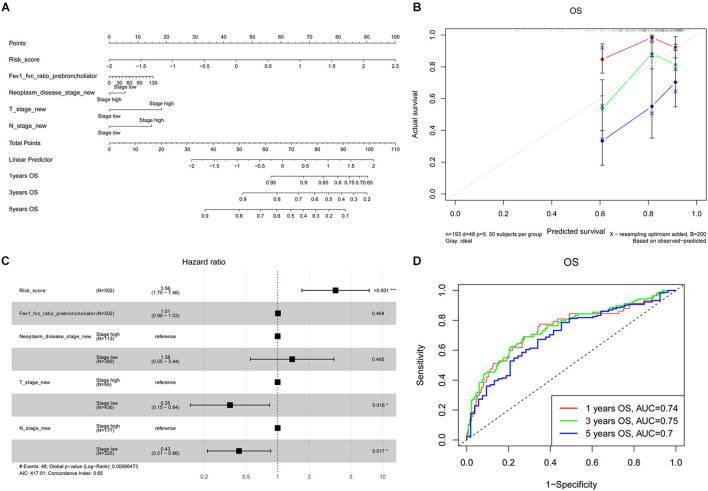
Development of prognostic NMGs nomogram for LUAD patients. **(A)** The nomogram for predicting 1-, 3-, and 5-year OS probabilities of LUAD patients. **(B)** Calibration plot of the established nomogram for predicting probabilities of 1-, 3-, and 5-year OS. **(C)** Multivariate Cox regression analysis of risk factors (including risk score, Fev1 Fvc ratio of prebroncholiator, neoplasm disease stage, T stage, and N stage). **(D)** The ROC curves of the nomogram for predicting 1-, 3-, and 5-year OS. OS, overall survival; ROC, receiver operating characteristic; T stage, tumor stage; N stage, lymph node stage.

As depicted in Supplementary Figure 1, the risk model was associated with Fev1 Fvc ratio of prebroncholiator, neoplasm disease stage, tumor (T) stage, and lymph node (N) stage (all *P* < 0.05). Meanwhile, it was unrelated with Fev1 Fvc ratio of postbroncholiator, age, long-distant metastasis (M) stage, adjuvant postoperative targeted therapy, and smoking history. Therefore, the nomogram was constructed based on the variables of risk score, Fev1 Fvc ratio of prebroncholiator, neoplasm disease stage, T stage, and N stage to predict the OS of patients with LUAD (Figure 4A). The concordant index (C-index) of the nomogram model to predict OS was 0.65 and the calibration curves of the 1-, 3-, and 5-year survival prediction were all close to the ideal performance of Figure 4B, exhibiting good predictive accuracy. Subsequently, these four variables (Fev1 Fvc ratio of prebroncholiator, neoplasm disease stage, T stage, and N stage) and risk score were selected for multivariate Cox regression analysis. Risk score was independently associated with a worse survival (HR = 3.56, 95% CI = 1.70–7.46, *P* < 0.001); on the contrary, low T stage (HR = 0.35, 95% CI = 0.15–0.84, *P* = 0.018) and low N stage (HR = 0.43, 95% CI = 0.21–0.86, *P* = 0.017) were significantly associated with better OS (Figure 4C). Then, the AUC for the 1-, 3-, and 5-year survival of the constructed nomogram were 0.74, 0.75, and 0.70, respectively (Figure 4D), indicating that the model was stable and robust for predicting the survival of patients with LUAD.

### Biological Processes in the High-Risk and Low-Risk Subgroups

**FIGURE 5:**
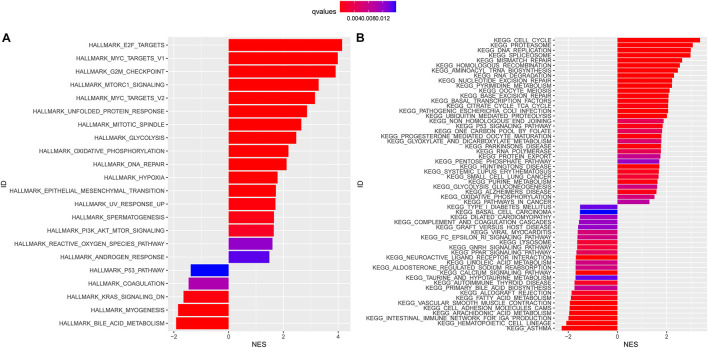
HALLMARK **(A)** and Kyoto Encyclopedia of Genes and Genomes (KEGG) **(B)** enrichment analysis in the high-risk and low-risk subgroups. The enriched items were selected with a corrected q-value 0.01; the length and color of the bar represent the absolute value of NES and the q-values, respectively. NES: normalized enrichment score.

HALLMARK enriched results revealed that the E2F targets, MYC targets, DNA repair, and G2M checkpoint signaling pathways were highly enriched in the high-risk subgroups (*P* < 0.05, Figure 5A). In other words, overexpression of the genes in the pathways mentioned above was positively correlated with the risk score. Meanwhile, we conducted the KEGG pathway analysis and found that NMGs were involved in the cell cycle, proteasome, and DNA replication signaling pathways (Figure 5B). Interestingly, both HALLMARK and KEGG analyses indicated that NMGs were enriched in metabolism-related signaling pathways such as glycolysis and oxidative phosphorylation signaling pathway in the high-risk subgroups.

### Genomic Mutations and the Immune Microenvironment in the High-Risk and Low-Risk Groups

**FIGURE 6:**
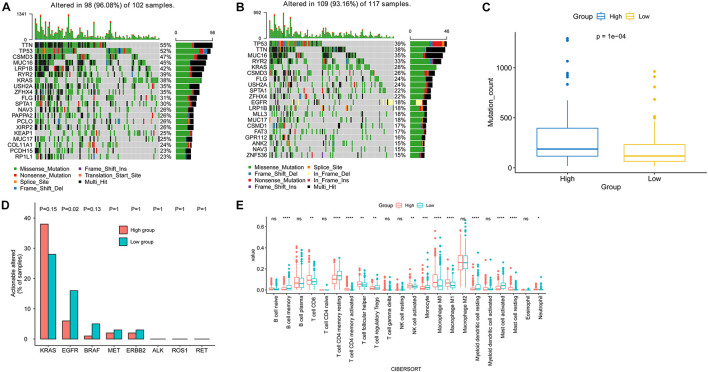
Genomic mutations and tumor microenvironment analysis in the high-risk and low-risk subgroups. The top 20 most frequently mutated genes in high-risk **(A)** and low-risk **(B)** subgroups. **(C)** Comparison of mutation count between the two risk subgroups. **(D)** Comparison of the prevalence of actionable genes in LUAD between two risk subgroups. **(E)** Comparison of immune cell infiltration between two risk subgroups.

The top 20 most frequently mutated genes in the high-risk and low-risk groups are shown in Figures 6A,B. The mutation count in the high-risk group was significantly higher than that in the low-risk group (Figure 6C). In addition, by comparison the prevalence of actionable alterations in LUAD between two groups, we found that there were more patients in the low-risk group having actionable *EGFR* alterations (Figure 6D).

Meanwhile, analysis of the tumor-infiltrated cells of TCGA-LUAD indicated the difference in immune cell infiltration between the high-risk and low-risk subgroups. The low-risk group had a significantly higher abundance of memory B cells, resting CD4 memory T cell, regulatory T cell (Tregs), monocyte, resting myeloid dendritic cell, and activated mast cell. On the other hand, the level of CD8 T cell, activated CD4 memory T cell, helper follicular T cell, activated NK cell, M0 macrophage, M1 macrophage, and resting mast cell in the high-risk group were higher than that of the low-risk group (Figure 6E).

### Genetic Alteration in Nuclear Mitochondrial Genes

**FIGURE 7:**
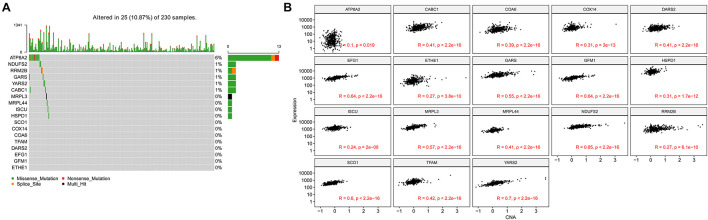
Genetic alteration and copy number alterations (CNAs) of the 18 genes in LUAD samples. **(A)** Genetic alteration percentage of 18 genes. **(B)** Correlation of each 18 genes mRNA expression level and linearized copy-number alteration.

In the TCGA-LUAD cohort, 10.87% of patients have genetic alterations among 18 NMGs, of which missense mutation accounts for the most prevalent type. The most prevalent altered gene is *ATP8A2* (6%), whereas other genes were rarely altered (Figure 7A). In addition, we found the mRNA expression of 18 genes was positively correlated with their relative linear copy number alteration (CNA) value, especially *EFG1, GFM1, NDUFS2, SCO1*, and *YARS2* (Figure 7B).

### The Therapeutic Benefit of the Risk Score Value

Pathway enrichment analysis showed that overexpression of genes in the DNA repair pathway was positively correlated with risk score, which suggests that a higher risk score indicated an increased ability of DNA repair. Thus, we speculate that the patients in the high-risk group exhibited a weaker response to radiation therapy compared with the low-risk group. To confirm our hypothesis, the patients who received radiation therapy (*N* = 61) from the TCGA cohort were separated into a high-risk and low-risk group based on the median risk score, and the patients with higher risk scores exhibited significantly worse prognosis (*p* = 0.038) (Supplementary Figure 2), which indicated NMGs signature might serve as a potential indicator of patients with LUAD response to radiotherapy.

## Discussion

The pivotal roles of mitochondria in maintaining normal cell function as well as in cancer development are widely acknowledged. Dysfunction in some specific members of mitochondrial genes, especially *FH*, *SDH* family, and *IDH1/2*, have been demonstrated as the milestone event for the development of multiple cancers like gliomas (Hartmann et al., 2009), glioblastomas (Balss et al., 2008), acute myeloid leukemia (Paschka et al., 2010), pheochromocytoma (Astuti et al., 2001), paraganglioma (Niemann and Müller, 2000; Astuti et al., 2001), and papillary renal cell cancer (Tomlinson et al., 2002; Luo et al., 2020). However, the integrated role of NMGs in the prediction for the prognosis of lung cancer patients remains undefined.

In this study, we first identified 26 NMGs whose expression were correlated with LUAD patients’ survival. Among those genes, only seven genes (*ATP8A2*, *CABC1*, *TK2*, *ANT1*, *RRM2B*, *COX14*, and *ISCU*) were associated with a favorable outcome in LUAD patients. Although these genes were previously reported in some cancer types, their specific role in lung cancer is unclarified except for *ATP8A2* and *RRM2B*. *ATP8A2*, encoded ATPase Phospholipid Transporting 8A2, belongs to the P4-ATPase family that actively flips phosphatidylserine and phosphatidylethanolamine from the exoplasmic to the cytoplasmic leaflet of cell membranes to generate and maintain phospholipid asymmetry (Choi et al., 2019). As this gene involves neurite elongation and neuron survival, various genomic variants of ATP8A2 are identified in patients with neurological disorders (Guissart et al., 2020). Though the relationship between ATP8A2 and lung cancer is poorly investigated, a recent study found the alteration in its expression was associated with the prognosis of LUAD (Wang et al., 2020). *ATP8A2* was hypermethylated in tumor tissues and, therefore, poorly expressed in the tumor tissues compared with the normal tissues (Wang et al., 2020). The same as the previously published result by Wang et al. (2020), we found *ATP8A2* was associated with a better prognosis in LUAD. *RRM2B*, which has been widely recognized for its important role in maintaining genomic stability, was widely overexpressed in many types of tumors. However, a previous study suggested the distinct roles of *RRM2B* in tumor progression: on one hand, its amplification was related to worse outcomes in breast cancers (Chae et al., 2016; Iqbal et al., 2021); on the other hand, *in vitro* analysis found its overexpression could inhibit the proliferation of lung cancer cells by the regulation work of FOXO3 (Cho et al., 2014). The latter result was also supported by our results as *RRM2B* was significantly associated with better survival in LUAD patients. Meanwhile, 18 NMGs significantly correlated with unfavorable prognosis in LUAD patients were identified. Among them, *SCO1*, *HSPD1*, *IARS2*, *MRPL44*, *TFAM*, *NDUFS2*, *MRPL13*, *OPA1*, and *YARS* have been previously identified as oncogenes in lung cancer or other types of cancers, serving as unfavorable prognosis biomarkers individually (Telang et al., 2012; Lee et al., 2017; Sotgia and Lisanti, 2017; Di et al., 2019; Dunham-Snary et al., 2019; Liu et al., 2019; Witherspoon et al., 2019; Li et al., 2020; Zhang et al., 2020; Cai et al., 2021). However, it is noteworthy that nearly all these findings were established and assessed *in vitro*, and all these NMGs were analyzed separately instead of integrating. Our novel identified unfavorable prognosis predictors, including *GFM1*, *KARS*, *DARS2*, *GARS*, *DLP1*, *MRPL12*, *COA6*, *SCO1*, and *C10ORF* have not been functionally analyzed previously. We are the first study to confirm their association with the progression of LUAD patients. Meanwhile, previous research found *COA6*, an OXPHOS complexes assembly factor, negatively correlated with vimentin levels, which was suggested as a favorable predictor for cancer prognosis (Cruz-Bermúdez et al., 2019). On the contrary, our findings suggested its association with unfavorable survival in LUAD patients. Furthermore, *MRPL44* and *MRPL12* were both members of mitochondrial ribosomal proteins, whose abnormal expression was found associated with the tumorigenesis and development of lung cancer (Huang et al., 2020). Glycolysis is a dominant metabolism for cancer cells to produce energy, even in the presence of oxygen. Research has demonstrated that a higher glycolytic rate in tumor cells was shown to promote resistance to chemotherapeutics (Ganapathy-Kanniappan and Geschwind, 2013). On the contrary, it has been revealed that mitochondrial OXPHOS is also utilized by cancer cells. Meanwhile, some evidence suggests that OXPHOS contributes to cancer metastasis (Viale et al., 2015; Yu et al., 2017; Rao et al., 2019). In the present study, glycolysis and oxidative phosphorylation signaling pathways were enriched in the high-risk subgroup in HALLMARK and KEGG enrichment analyses. These signaling pathways may play important roles in tumorigenesis and the progression of LUAD. Nowadays, the treatment landscape of NSCLC includes target therapy, immunotherapy, and combination therapy, which is mainly decided by the patients’ driver genes status, especially *EGFR* and *ALK* (Grant et al., 2021). Interestingly, our findings showed that LUAD patients with high or low NMGs signature may have distinct treatment choices as the differences in the tumor microenvironment and driver alterations. LUAD patients with low NMGs signature have not only improved outcomes but also significantly higher prevalence of EGFR alterations, which may contribute to the response to *EGFR* tyrosine-kinase inhibitors. On the contrary, patients with high NMGs have more inflamed immune features, including higher mutation counts, high presence of immune-prone TILs (such as CD8 positive T cells, activated CD4 memory T cell and NK cell), and lower presence of immunosuppressive TILs (such as Treg cells). Previous studies have found the tumor-infiltrated CD8 positive T cells prior to immune checkpoint inhibitors could serve as an indicator for the response of anti-PD-1/L1 therapy (Tumeh et al., 2014; McDermott et al., 2018). Based on these features, it is indicated that LUAD patients with high NMGs signature may benefit more from immune checkpoint inhibitors (Lucibello et al., 2021). In concordance with a more inflamed tumor microenvironment, tumor mutation counts (also known as tumor mutation burden, TMB) level was significantly higher in the high NMGs signature than in the low group, which has already been granted as an effective biomarker for selecting patients who may respond to pembrolizumab by FDA (Marcus et al., 2021).

There were some limitations in our study. Although with ample validation, the signature was established and validated based on the public datasets. Further validation in a local cohort using prospective clinical samples and data is merited. Though we found the association between NMGs signature and tumor microenvironment or driver gene alterations (especially EGFR), which may affect the decision-making for clinical management for LUAD patients, the validation of the indication that the difference in the efficacy of immune checkpoint inhibitor and targeted therapy on patients with high- or low-risk signature is worthy to explore in further study, as well as the underlying regulatory mechanism *in vivo* or *in vitro*. Based on the above limitations, we have designed a local LUAD cohort from our hospital and performed testing of the involved NMGs based on real-time PCR to further verify the results of this study.

In conclusion, we conducted an integrative analysis of the role of NMGs in the prognosis of LUAD and successfully established a robust and stable prognosis predicting signature with sufficient validation. Except for prognostic function, this established signature could also distinct LUAD patients who may have more clinical benefits from radiotherapy, targeted therapy, and immune checkpoint inhibitors, which may give clinicians more decision-making help to manage patients.

## Data Availability Statement

The datasets presented in this study can be found in online repositories. The names of the repository/repositories is cBioPortal and accession number(s) can be found in the article/Supplementary Material.

## Author Contributions

WM, XZ, and WD: conception and design, data analysis and interpretation (statistical analysis), and manuscript writing and revision. JZ, WL, DS, and JY: data acquisition and manuscript writing. All authors read and approved the final manuscript.

## Conflict of Interest

The authors declare that the research was conducted in the absence of any commercial or financial relationships that could be construed as a potential conflict of interest.

## Publisher’s Note

All claims expressed in this article are solely those of the authors and do not necessarily represent those of their affiliated organizations, or those of the publisher, the editors and the reviewers. Any product that may be evaluated in this article, or claim that may be made by its manufacturer, is not guaranteed or endorsed by the publisher.

## Footnotes

1
https://www.mitomap.org/MITOMAP

2
https://www.gsea-msigdb.org/gsea/msigdb/index.jsp

## Supplementary Material

Supplementary Figure 1 Comparison of clinical characteristics between the high-risk and low-risk groups.

Supplementary Figure 2 The therapeutic benefit of the risk score value. Kaplan-Meier OS curves for patients who received radiation therapy assigned to high- and low-risk groups in the TCGA cohort.
