# Supplementary figures and images for: A Novel Mitochondrial-Related Nuclear Gene Signature Predicts Overall Survival of Lung Adenocarcinoma Patients
Source: Front Cell Dev Biol. 2021 Oct 25;9:740487. doi: 10.3389/fcell.2021.740487 (PMC8573348; doi:10.3389/fcell.2021.740487)

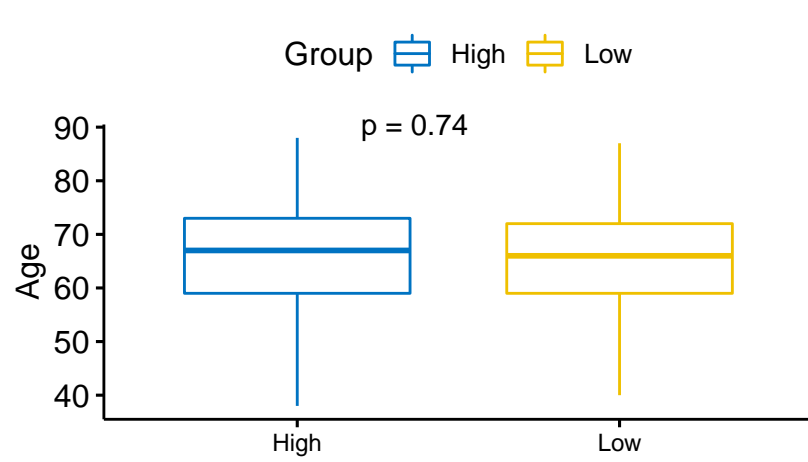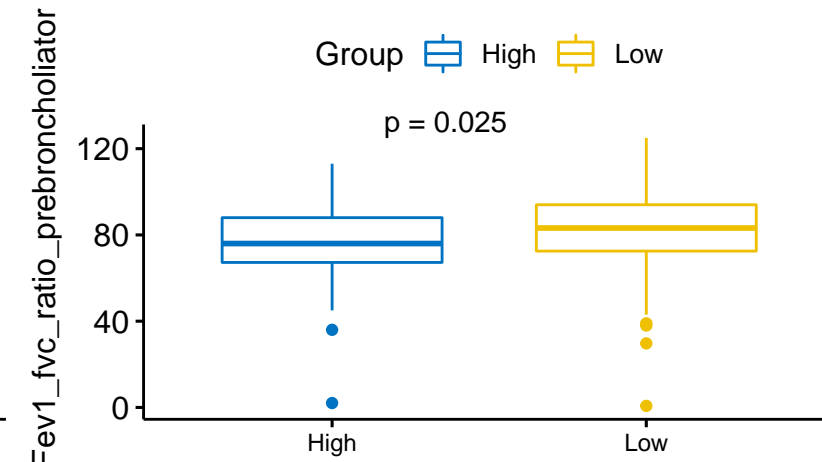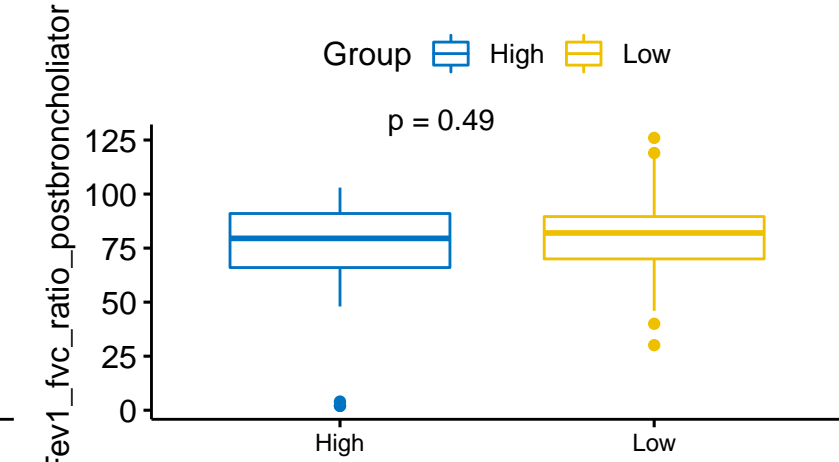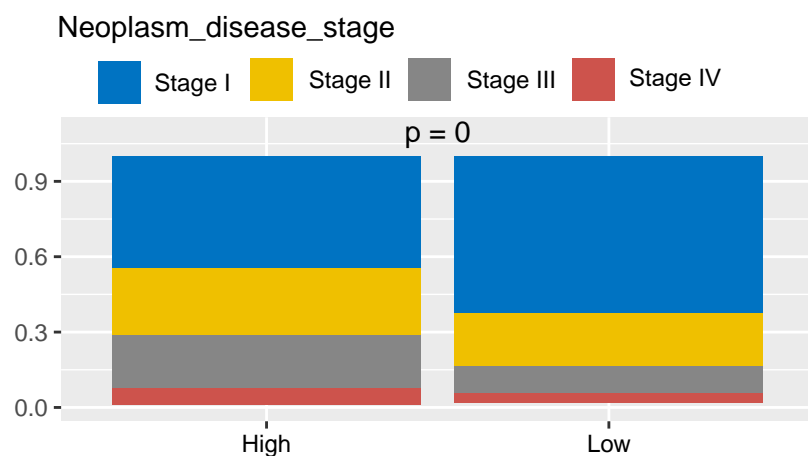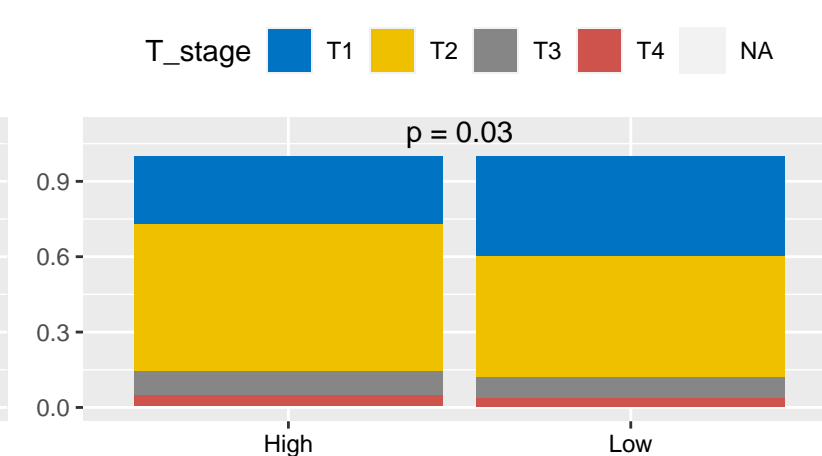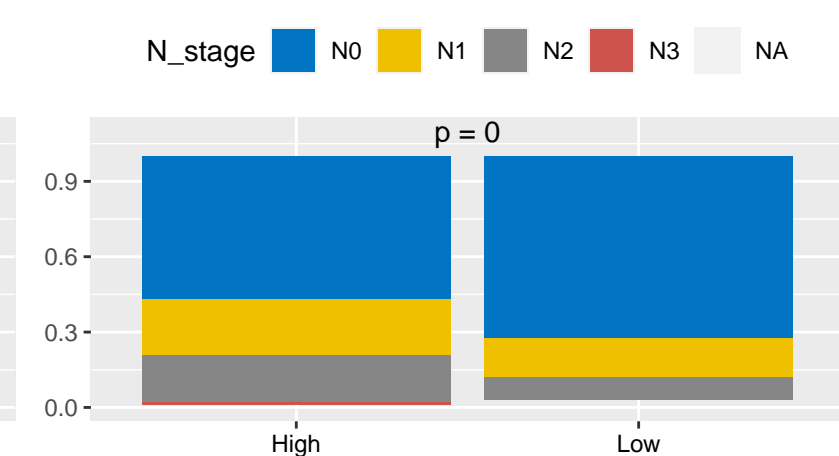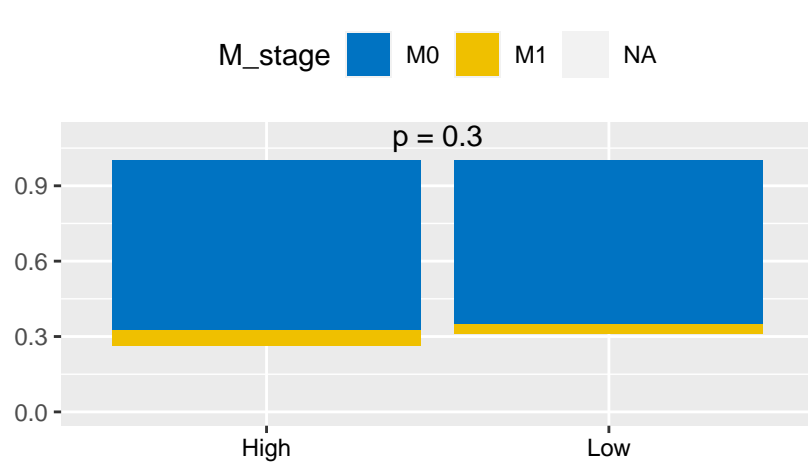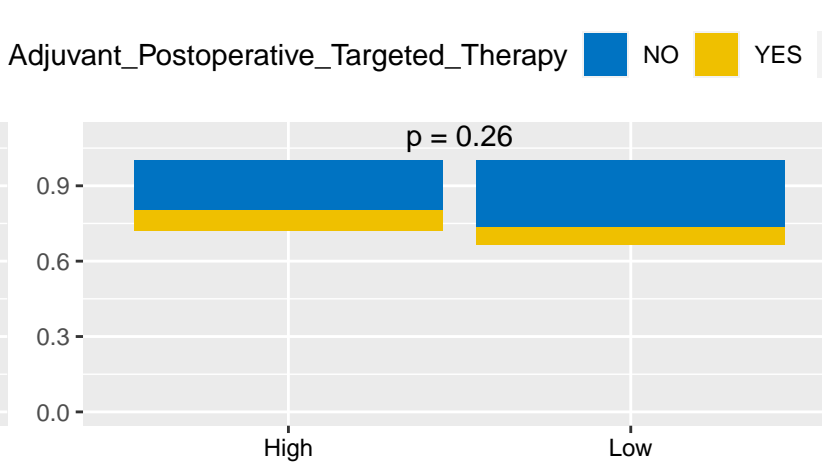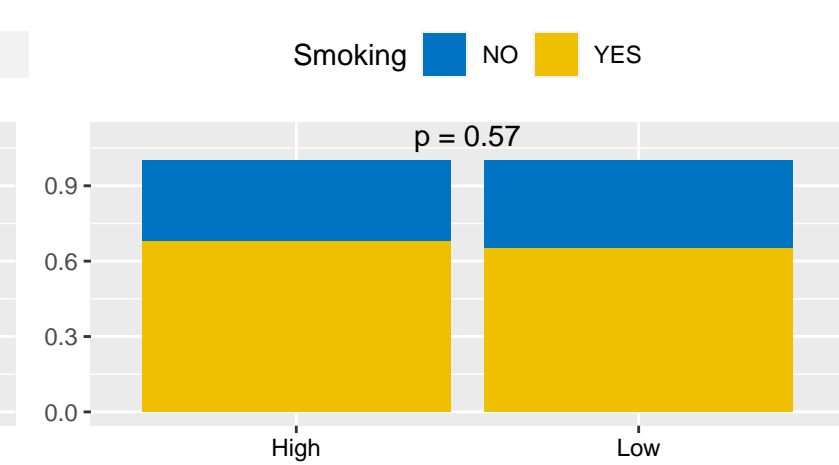

Supplement: Supplementary Figure 1 — Comparison of clinical characteristics between the high-risk and low-risk groups. [file Data_Sheet_1.PDF]

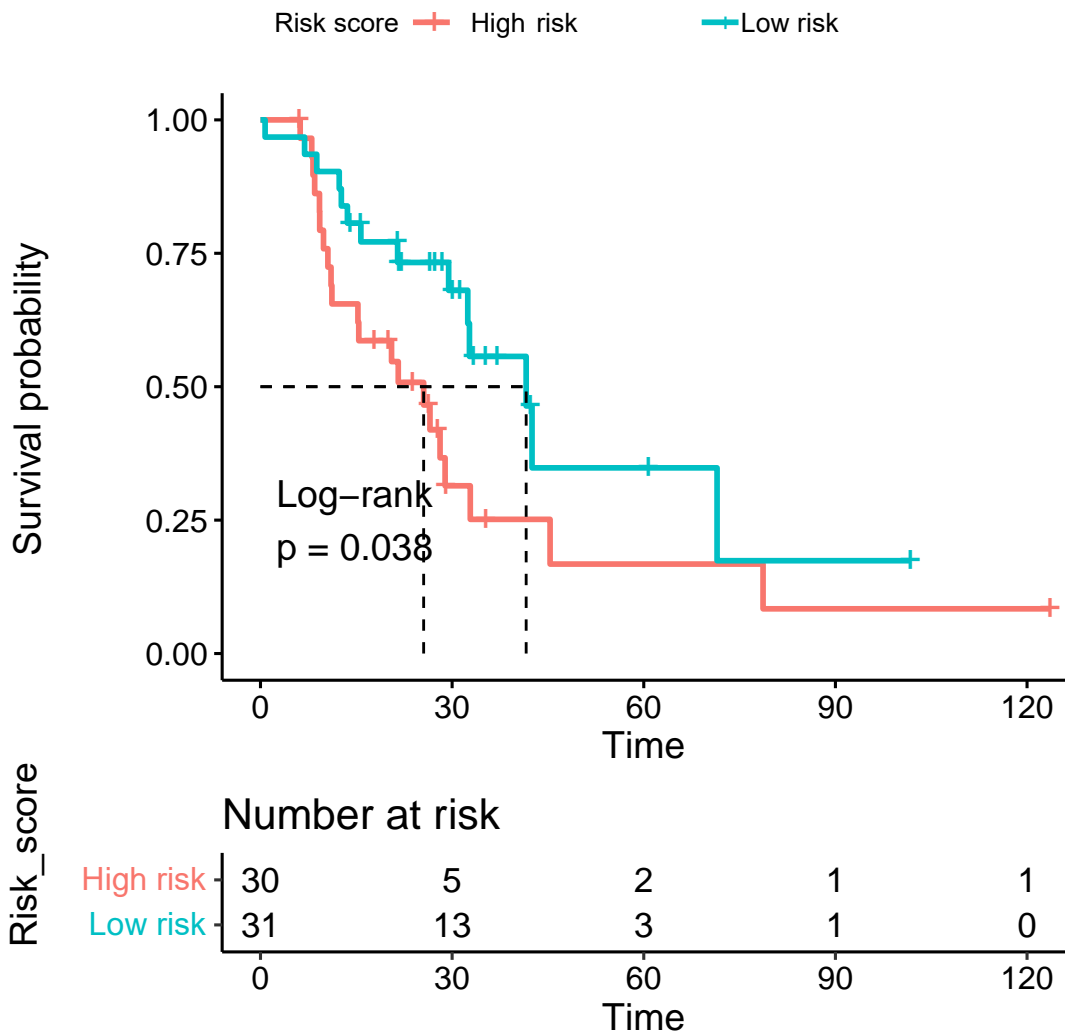

Supplement: Supplementary Figure 2 — The therapeutic benefit of the risk score value. Kaplan-Meier OS curves for patients who received radiation therapy assigned to high- and low-risk groups in the TCGA cohort. [file Data_Sheet_2.PDF]
